# Spruce-derived extracellular vesicles (SDEVs) improve hair loss by activating the proliferation of dermal papilla cells

**DOI:** 10.1371/journal.pone.0358041

**Published:** 2026-11-06

**Authors:** Kunchi Zhang, Ying Cao, Zitong Wang

**Affiliations:** Shanghai Key Laboratory of Molecular Imaging, Shanghai University of Medicine and Health Sciences, Shanghai, China; Shanghai Jiao Tong University Medical School Affiliated Ruijin Hospital, CHINA

## Abstract

Hair loss is a global health problem, and current conventional treatments, despite their effectiveness in delaying hair loss and improving hair growth, suffer from significant side effects and limitations in individualized treatment. In recent years, the application of novel nanotechnology in dermatology and regenerative medicine has gained attention. In this context, researchers explored the potential of applying Spruce-Derived Extracellular Vesicles (SDEVs) to hair loss intervention. Through in vitro experiments and in vivo animal models, it was found that Spruce-Derived Extracellular Vesicles (SDEVs) could improve hair follicles by activating the proliferation of Dermal papilla cells (DPCs) to intervene in hair loss. Spruce-Derived Extracellular Vesicles (SDEVs) significantly improved hair weight, thickness, and luster, and reduced the inflammatory response associated with hair loss compared with conventional treatments. These findings provide new ideas for hair regrowth treatments and demonstrate the potential therapeutic value of Spruce-Derived Extracellular Vesicles (SDEVs) in hair loss prevention.

## 1. Introduction

Hair loss is a common dermatological and cosmetic concern that can susbstantially affect quality of life.With the development of science and technology, modern medicine has made significant progress in addressing hair loss, mainly through medication and surgery, but these methods either have side effects or require long-term adherence and are costly. Therefore, increasing attention has been directed toward alternative or adjunctive approaches. In this context, plants and their extracts show great potential for use in hair loss prevention and treatment due to their high safety, low side effects, and significant efficacy. In recent years, natural plant-derived anti-hair loss products have gradually become a research hotspot [1].

Spruce is a type of coniferous tree in the family Pinaceae, commonly distributed in the boreal and temperaete regions of the Northern Hemisphere. It is known for its strong growth capacity and good resistance [2,3]. In Chinese traditional medicine, Pinaceae plants have been reported to have certain anti-hair-loss effects. However, there are insufficient studies on Spruce and hair loss, and further exploration of its mechanisms and potential applications is still needed [4,5]. In studies on follicle-associated hair growth, dermal papilla cells (DPCs) play a key role in the growth cycle of the hair follicle and regulate this process by secreting a variety of growth factors and signaling molecules, such as fibroblast growth factor (FGFs), platelet-derived growth factor (PDGF), and vascular endothelial growth factor (VEGF), which are involved in cell preliferation, epithelial-mesenchymal interactions, and perifollicular angiogenesis [6,7]. These growth factors regulate the growth cycle of hair follicles [8,9]. When the DPCs function is impaired, hair follicles may enter the resting phase prematurely, leading to hair loss [10–12]. The growth of hair follicles can be regulated by modulating the function of DPCs, which in turn promotes hair growth.

Plant-derived extracellular vesicles (PDEVs) are nano-sized vesicles secreted by the cells themselves, which contain a wide range of intracellular biomolecules and proteins [13]. Because of their ability to directly reflect the internal structure and function of plants, as well as their potential ability to transmit information within the organism, they are considered as a new way to study the effects of plants on diseases in the organism [14]. Compared with conventional crude plant extracts, PDEVs may represent a distinct bioactive fraction for mechanistic studies, although their isolation and characterization still require rigorous standardization. In recent years, increasing attention has been paid to PDEVs because of their biocompatibility, low immunogenicity, and potential utility as natural bioactive agents or delivery vehicles [15–17]. Based on the above findings, this study collected and characterized Spruce-Derived Extracellular Vesicles (SDEVs) and examined their effects on hair growth and on hair follicle formation and regeneration by studying their proliferative effects on Dermal papilla cells (DPCs). This study provides a basis for the application of Spruce-Derived Extracellular Vesicles (SDEVs) to improve hair loss.

## 2. Materials and methods

### 2.1. Materials

DMEM medium, fetal bovine serum (FBS), penicillin-streptomycin (double antibiotic), and 0.25% trypsin were purchased from Reis Biotechnology (Shanghai) Co., Ltd. Phosphate-buffered saline (PBS, 1×) was purchased from Wuhan Xavier Biotechnology Co., Ltd. BCA Protein Quantification Kit (Enhanced) and Neutral Formalin Fixative (10%) were purchased from Adamas-life Sciences Co., Ltd. Minoxidil and Tolbutamide were purchased from Shanghai Yuanye Biotechnology Co., Ltd. Cell Counting Kit-8 (CCK-8) was purchased from MedChem Express Co., Ltd. Minoxidil application was purchased from Gold Pharmaceuticals. Human VEGF-A ELISA kit was obtained from Enzyme Immuno Co., Ltd. LAT Mouse Anesthetic (Tribromoethanol) was obtained from Layette. All other reagents were purchased from Greagent. T25 cell culture flasks were purchased from Wuxi NEST Biotechnology Co., Ltd. Six-well and 96-well cell culture plates, as well as 15- and 50- mL centrifuge tubes, were purchased from Corning. In addition, 2000-mL conical flasks and 1-mL sterile disposable pipettes were obtained from Titan Pharmaceuticals, Inc. Filters with pore sizes of 0.22 μm and 0.45 μm were purchased from Millipore. Seventy-milliliter centrifuge bottles were purchased from Beckman Coulter, Inc.

### 2.2. Preparation of SDEVs

Spruce needles were weighed, cleaned with sterile water, added with an appropriate amount of pre-cooled PBS solution, and extracted using a screw juicer. The plant residue was discarded and the filtrate was collected, transferred to a 50 mL centrifuge tube and centrifuged at 2000 × g for 15 min at 4 °C, and the supernatant was collected and centrifuged at 120,000 × g for 90 min at 4°C. The supernatant was continued to be collected using a floor-type ultracentrifuge (Beckman Coulter, Type 45 Ti rotor head) and discarded. After centrifugation for 30 min, the supernatant was continued to be collected by centrifugation at 120,000 × g for 90 min at 4 °C using a floor-type ultracentrifuge (Beckman Coulter, Type 45 Ti rotor head), the upper layer of the clear liquid was discarded, and the precipitate was resuspended with PBS. The resuspended liquid was filtered through a 0.22 μm filter, and the filtrate became the solution of SDEVs, which was stored at 4 °C for spare use.

### 2.3. Characterization of physicochemical properties of SDEVs

Pipette 5 μL of SDEVs, drop it on the copper mesh, and stain it with phosphotungstic acid. Finally, the copper mesh was removed, excess liquid was absorbed with filter paper, dried and observed under a transmission electron microscope, photographed and the pictures were saved. The samples to be tested were diluted with PBS at 1:100 and placed on ice for backup. Inside the nanoparticle tracking analysis technique instrument, 5 mL of purified water and 5 mL of PBS buffer were injected sequentially to clean the sample cell. Inject 1 mL of standard sample solution and set the parameters. Inject 2 mL of diluted samples to be tested, and inject the samples for detection to obtain the concentration and particle size information of SDEVs.

### 2.4. CCK8 study of SDEVs on DPCs proliferation

Cell culture was performed using complete medium, formulated with a high-sugar version of DEME medium, 10% FBS, and 1% Antibiotic-Antimycotic. Cells were inoculated in 96-well cell culture plates at a density of 1 × 10^4^ cells per well and cultured for 24 h. The cells were then incubated with the SDEVs. SDEVs were added, and a blank control group without EVs, an apoptosis group without cells and samples, and a positive control group with 10 μg/mL minoxidil were set up, with 5 replicate wells in each group. After 48 hours of incubation, the supernatant was discarded and washed with PBS, and 220 μL of freshly prepared CCK8 solution was added to each well. The wells were incubated at 37°C for 4 hours, and the absorbance values were read at 450 nM on an enzyme meter.

### 2.5. CCK8 study of changes in DPCs’ potassium ion channels

Cells were digested and inoculated into 96-well cell culture plates at a density of 2 × 10^4^ cells per well for 24 h. After washing with PBS, freshly prepared 1.5 mM potassium ion antagonist Tolbutamide was added to 100 μL per well. After incubation for 4 h, the supernatant was discarded, SDEVs were added, and a blank group, a negative control group, and a positive control group for minoxidil were set up, with 5 replicate wells in each group. After 24 hours of incubation, the supernatant was discarded and washed with PBS, and 220 μL of freshly prepared CCK8 solution was added to each well. The wells were incubated at 37°C for 4 hours, and the absorbance was measured at 450 nM with an enzyme meter.

### 2.6. EdU cell proliferation assay

DPCs were cultured in DPCs-specific complete medium and seeded into confocal dishes at a density of 5 × 10^4^ cells per dish. After 12 h of incubation, the culture medium was replaced with serum-free DPCs-specific medium and the cells were maintained for an additional 12 h to achieve cell cycle synchronization. Subsequently, the medium in the treatment group was supplemented with SDEVs at a final concentration of 600 μg/mL, while the control group received fresh DPCs-specific medium only. At 24 h and 48 h post-treatment, cell proliferation was assessed using the BeyoClick™ EdU Cell Proliferation Kit according to the manufacturer’s protocol. Cells were incubated with 10μM EdU for 2 h at 37 °C, fixed with 4% paraformaldehyde for 15 min, and permeabilized with 0.5% Triton X-100 for 10 min. EdU detection was performed via click chemistry reaction, followed by nuclear counterstaining with DAPI. Fluorescence images were captured using a confocal microscope with consistent acquisition parameters across all groups. Three independent biological replicates were prepared for each group to minimize experimental variability.

### 2.7. Ki-67 immunofluorescence assay

DPCs were cultured in DPCs-specific complete medium and seeded into confocal dishes at a density of 5 × 10⁴ cells per dish. After 12 h of incubation, the culture medium was replaced with DPCs-specific serum-free medium for an additional 12 h to achieve cell cycle synchronization. Subsequently, small extracellular vesicles (SDEVs) were supplemented to the medium of the treatment group at a final concentration of 600 μg/mL, while only fresh DPCs-specific medium was renewed for the control group. At 24 h and 48 h post-treatment, cell proliferation was assessed via Ki-67 immunofluorescence staining. Briefly, cells were first fixed with 4% paraformaldehyde for 15 min and permeabilized with 0.5% Triton X-100 for 10 min. Then 5% BSA blocking solution prepared in 0.1% Triton X-100 was added, and blocking was performed at room temperature for 1 h. Afterwards, Ki-67 primary antibody working solution (diluted 1:250 in 0.1% Triton X-100) was added and incubated overnight at 4 °C. Fluorescent secondary antibody working solution (diluted 1:500 in PBS) was then added and incubated for 1 h at room temperature in the dark, followed by DAPI counterstaining for 5 min. Fluorescence images were captured using a confocal microscope with consistent acquisition parameters across all groups. Three independent biological replicates were prepared for each group to minimize experimental variability.

### 2.8. Effects of SDEVs on hair growth in mice

All animal experiments were performed in strict accordance with the guidelines and protocols approved by the Animal Ethics Committee of Shanghai University of Medicine and Health Sciences (No.2018-GZR-19-1700007). Healthy C57BL/6 mice with normal hair were selected, and after one week of routine feeding, the hair on the back of the mice was removed with a shaver and then depilated with depilatory cream. Mice with smooth and undamaged skin in the depilated area were divided into a blank group, a negative control group, a Minoxidil-positive control group and a sample group, with 3 mice in each group. The drug was administered 48 hours after depilation: the blank group was left untreated; the negative control group was given 10% propylene glycol mixed with 5% ethanol; the Minoxidil group was given 2% Minoxidil rub; and the sample group was given SDEVs. 200 μL of the drug was applied to the skin of the mice in all the groups for three weeks, and the growth of the hair in the depilated areas of the mice was continuously recorded.

### 2.9. HE staining of mouse skin tissue

Fresh skin tissues were fixed in 4% paraformaldehyde solution for not less than 24 hours. After dehydration and embedding, the wax-impregnated tissues were transferred to the embedding machine. The wax blocks were trimmed and sectioned on a paraffin sectioning machine at a thickness of 4 μm. Finally, the sections were stained with HE, sealed with neutral gum and photographed under a microscope to document the morphology of the tissues.

### 2.10. Proteomic analysis of Spruce-Derived Extracellular Vesicles

Proteins were extracted from SDEVs using a phenol-based protocol. Samples were lysed in BPP buffer containing 100 mM EDTA, 50 mM borax, 50 mM vitamin C, 30% sucrose, 100 mM Tris base, 1% Triton X-100, 1%−5% PVPP, and 5 mM DTT, and protease inhibitors, followed by grinding at −40 °C for four cycles. An equal volume of Tris-saturated phenol (pH 8.0) was added, and the mixture was vortexed for 10 min and centrifuged at 12,000 × g for 20 min at 4 °C. Proteins were precipitated with five volumes of pre-cooled 0.1 M ammonium acetate in methanol. After centrifugation, the pellet was washed twice with 90% acetone, dissolved in 8 M urea containing protease inhibitors, and centrifuged again. Protein concentration was determined using BCA.

Protein digestion and peptide cleanup were performed using the iST Sample Preparation kit (PreOmics, Germany). Briefly, protein samples were mixed with 50 μL lysis buffer and incubated at 95°C for 10 min with agitation at 1000 rpm. After cooling, trypsin digestion buffer was added, and the samples were incubated at 37°C for 2 h at 500 rpm. Digestion was terminated with stop buffer, and peptides were desalted using the iST cartridge, eluted with elution buffer, vacuum-dried, and stored at −80°C until analysis.

For LC-MS/MS analysis, dried peptides were reconstituted in solvent A (0.1% formic acid in water) and analyzed on a Vanquish Neo UHPLC system (Thermo Fisher Scientific, MA, USA) coupled to a timsTOF HT mass spectrometer (Bruker Daltonik, Bremen, Germany). Peptides were separated on an AUR3-15075 C18 analytical column at 50 °C and 300 nL/ min using a 30 min gradient of 2.2%−44% solvent B over 21 min, followed by 44%−90% over 3 min, holding at 90% for 3 min, and re-equilibration at 4% for 3 min. DIA data were acquired in diaPASEF mode over an m/z range of 380.8–1170.8 using 82 × 10 Th precursor isolation windows, with collision energy linearly ramped from 59 to 20 eV according to ion mobility. Raw data were processed in Spectronaut 20 against the GP20250107YMB.fasta database with trypsin specificity, Carbamidomethyl on cysteine was specified as the fixed modification. Oxidation on methionine, Acetyl on protein N-term were specified as the variable modifications. Dynamic iRT was used for retention time calibration, and false discovery rates were controlled at 1% at the precursor peptide, and protein levels. Decoy generation was performed using the mutated strategy. Local normalization was applied in Spectronaut, and peptides passing the 1% Q-value cutoff were used for protein group quantification with the MaxLFQ method.

## 3. Results

The isolated SDEVs were characterized by transmission electron microscopy (TEM), nanoparticle tracking analysis (NTA), and protein quantification. TEM imaging revealed predominantly rounded or cup-shaped membrane-bound nanostructures with typical vesicular morphology (Fig 1). NTA showed that the particles had a mean diameter of 151.9 nm, indicating that the preparation was enriched in nanoscale vesicles within the operational size range generally assigned to small extracellular vesicles (Fig 2). The total protein concentration of the SDEV preparation was 3.537 mg/mL, confirming the presence of protein-containing material and providing a basis for dose normalization in subsequent experiments (Fig 3). Collectively, these findings demonstrate that the isolated sample contained nanosized particles with vesicle-like morphology and an appropriate size distribution.

**Fig 1:**
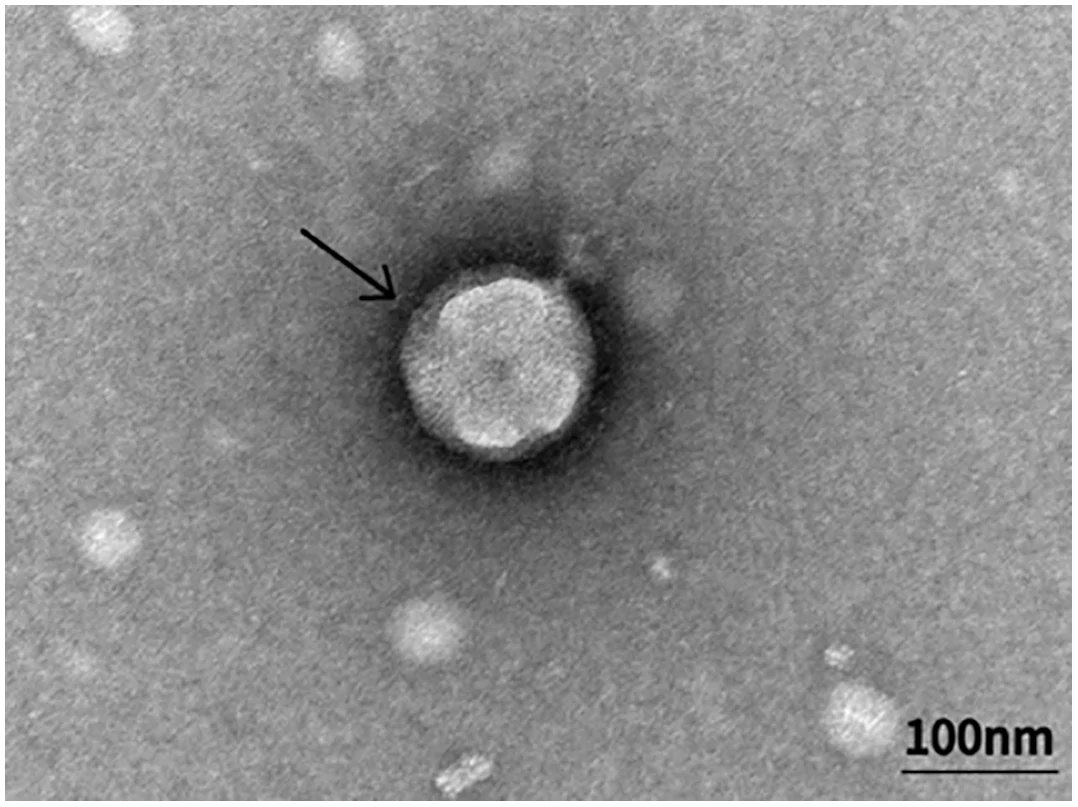
Morphology of Spruce EVs taken by TEM.

**Fig 2:**
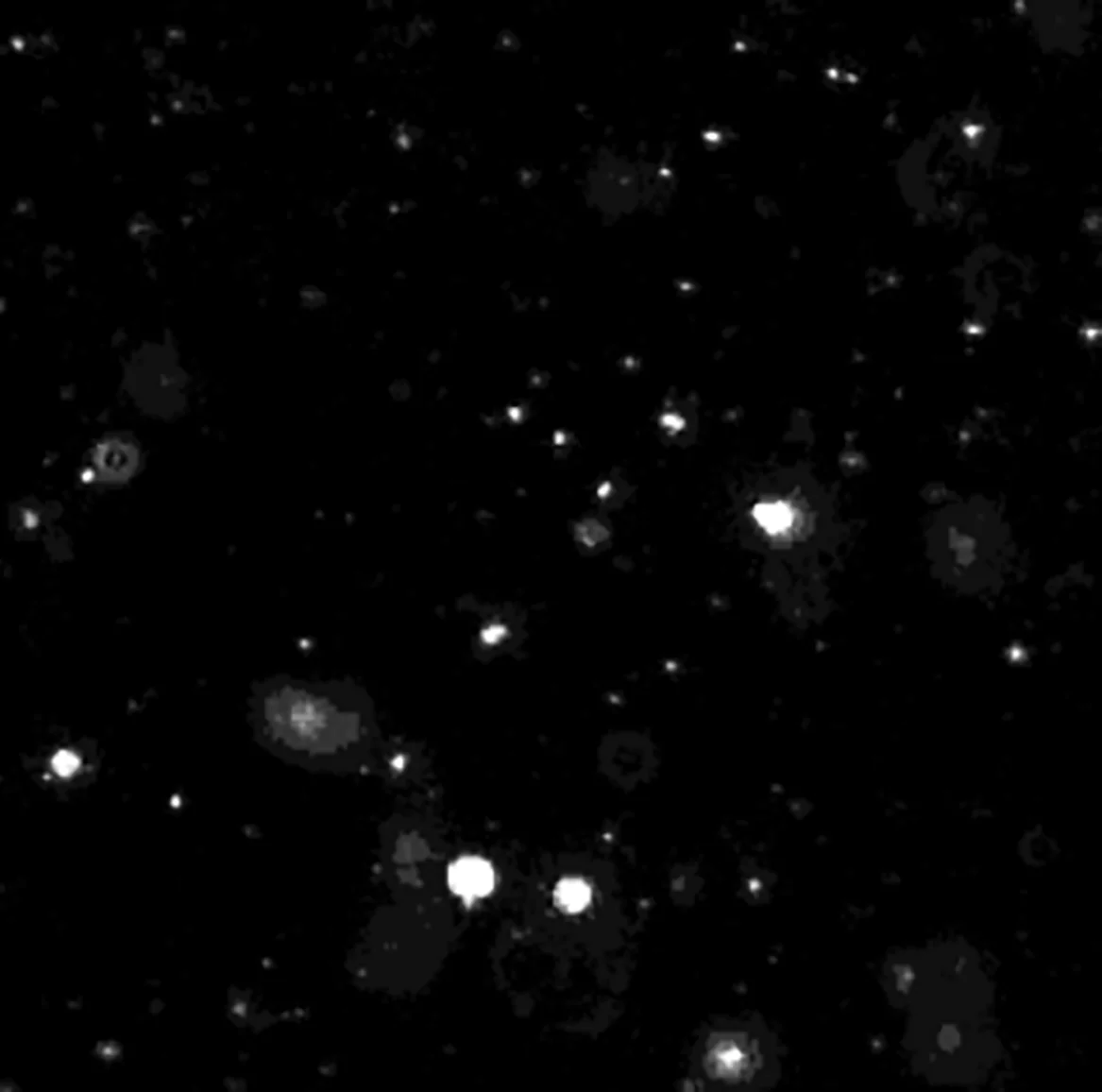
Particle distribution of Spruce EVs measured by NTA.

**Fig 3:**
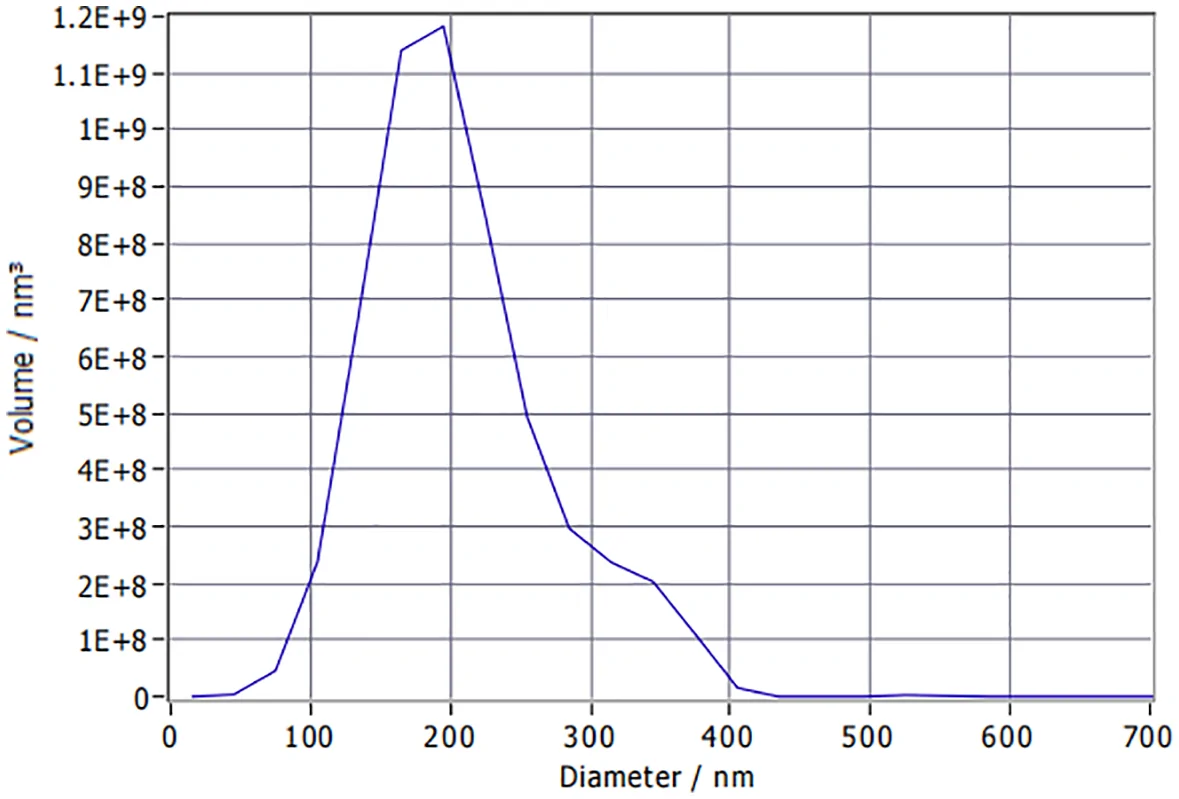
Particle size of SpruceEVs detectedby NTA.

The CCK-8 method was used to investigate the effect of different SDEVs protein concentrations on the viability of DPCs, and different SDEVs protein concentrations were used to incubate DPCs. 10 μg/mL of minoxidil and SDEVs significantly increased the cell survival rate of DPCs compared with the blank control, and the effect of higher-concentration SDEVs on cell survival was greater than that of Minoxidil. SDEVs with different protein concentrations had differences in cell survival and showed a dose effect, as shown in Fig 4. Based on the above results, the concentration that significantly affects cell survival can be selected for subsequent studies.

**Fig 4:**
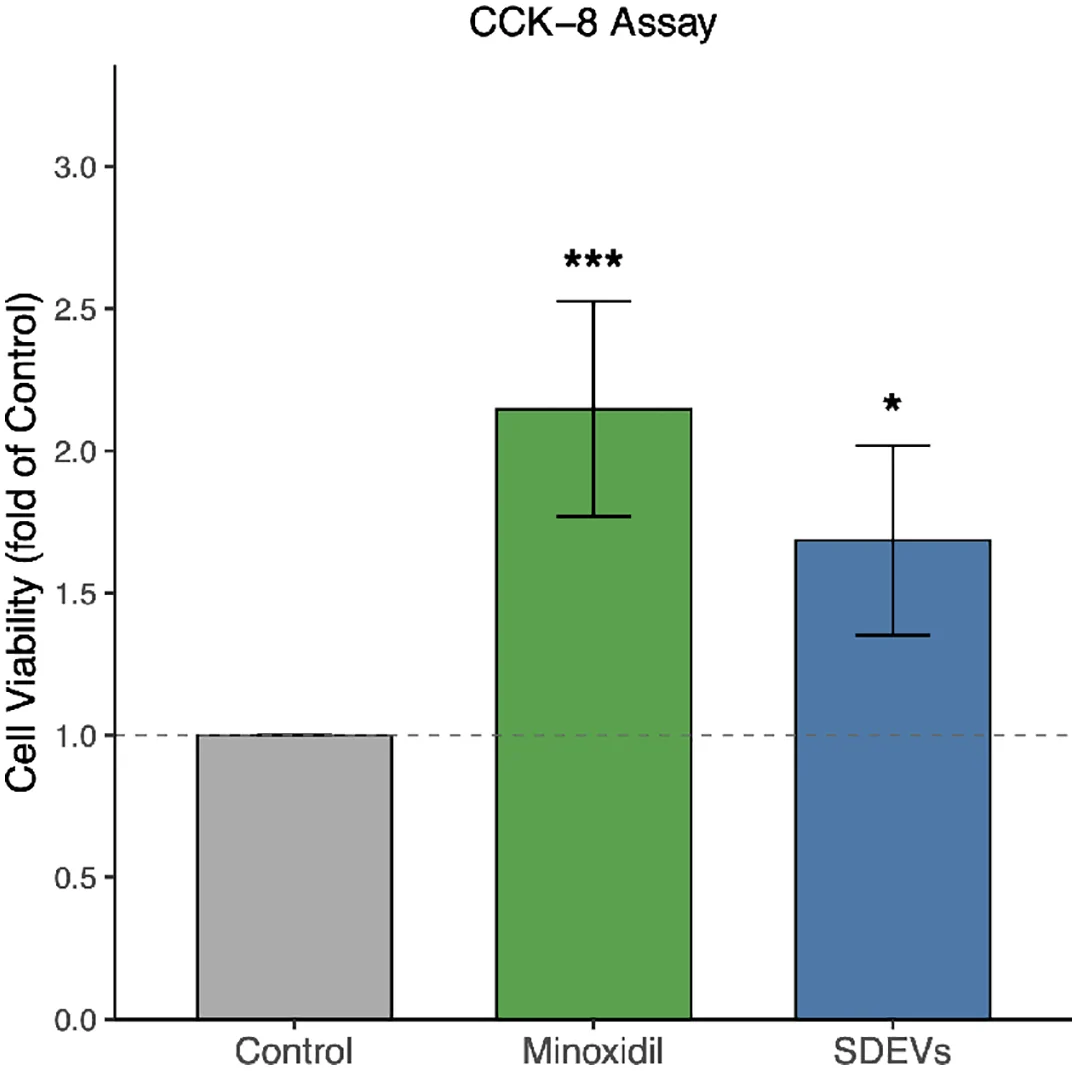
Effects of Spruce exosomes on proliferation of DPCs by CCK8 assay.

Tolbutamide, a potassium channel blocker, inhibits the proliferation of Dermal papilla cells (DPCs). After pretreatment of damaged cells with potassium antagonists, SDEVs were given to examine the effect of cell viability under different treatments. The CCK-8 assay was used to detect the effects of different protein concentrations of SDEVs on the viability of DPCs. After treatment with 1.5 mM Tolbutamide, cell viability decreased, and then both Minoxidil and SDEVs were given, cell viability increased. It was hypothesized that SDEVs could promote the proliferation of DPCs by activating potassium ion channels, and SDEVs had a dose-dependent promoting effect on DPCs viability, as shown in Fig 5.

**Fig 5:**
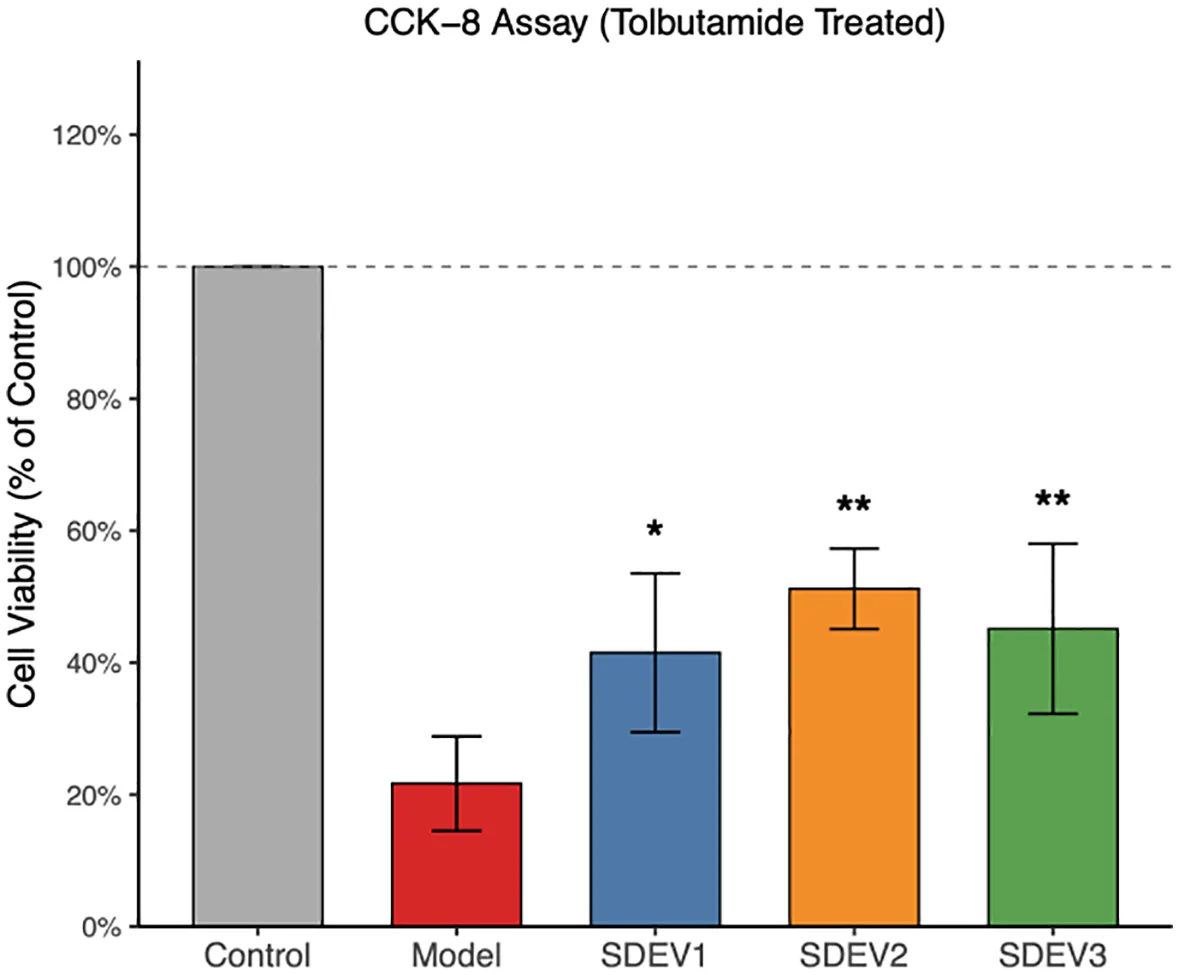
Effect of Spruce exosomes on proliferation of DPCs potassium channels detected by CCK8 assay.

EdU incorporation and Ki-67 immunofluorescence assays both showed that SDEVs treatment increased proliferation-related indices in DPCs. In the EdU assay, S-phase cells were labeled with Azide 594. DAPI was used to counterstain cell nuclei and determine total cell number. For Ki-67 immunofluorescence staining, cells were incubated with a rabbit monoclonal anti-Ki-67 antibody followed by an Alexa Fluor® 488-conjugated anti-rabbit IgG agment antibody. Ki-67-positive cells in the G1, S, G2, and M phases are shown in green, whereas quiescent G0-phase cells are Ki-67-negative. The control group received the same DPCs-specific medium without SDEVs. After 24 h of treatment, the proportion of EdU-positive cells increased from 20.0% to 23.5%, while the proportion of Ki-67-positive cells increased from 68.8% to 70.8%. After 48 h of treatment, the proportion of EdU-positive cells increased from 18.9% to 23.5%, and the proportion of Ki-67-positive cells increased from 73.9% to 80.0% (Fig 6). The increase in EdU positivity indicates that a greater proportion of DPCs entered the S phase and underwent DNA synthesis, whereas the increased Ki-67 positivity suggests an increased proportion of cells in an active cell-cycle state. The consistent direction of these two assays indicates that SDEVs may promote cell-cycle progression and proliferative activity in DPCs, with a more pronounced effect observed at 48 h. Compared with the CCK-8 assay, which primarily reflects cellular metabolic activity, the EdU and Ki-67 results provide more direct cellular evidence that SDEVs promote DPCs proliferation.

**Fig 6:**
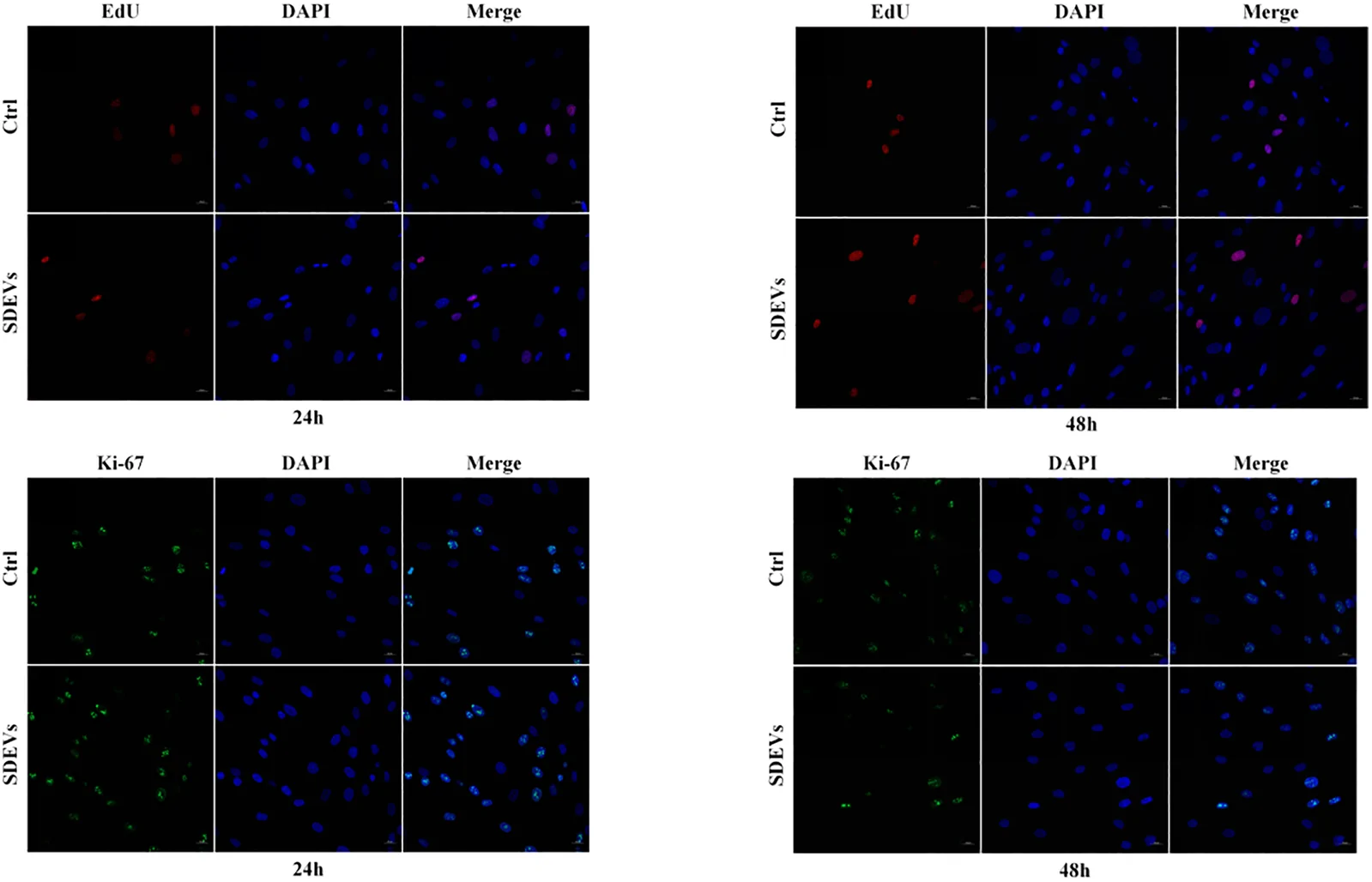
Representative confocal images of EdU incorporation and Ki-67 immunofluorescen ce staining in DPCs treated with 600 μg/mL SDEVs for 24 h and 48 h. Azide 594: red, excitation/emission maxima: 590/615 nm, DAPI: blue, excitation/emission maxima: 358/461 nm, Alexa Fluor® 488: excitation/emission maxima: 495/519 nm.

Hair growth on the back of mice in each group is shown in Fig 7. Hair growth in C57BL/6 mice is accompanied by the synthesis of melanin in the skin throughout the hair growth cycle, and the production of melanin in the skin is closely linked to the hair cycle, with the skin transforming from dark grey to black during the growth of the hair, and presenting a light pink color at the resting period. The skin at the hair removal site of mice (on the first day) appeared light pink. On the 7th day of administration, the site of the positive control group began to turn gray-black. On the 14th day of administration, the back of the mice of each group appeared to have centrally diffused growth of black glossy neonatal hair, and the effect of the positive control group (treaede with minoxidil) and the SDEVs group was significantly higher than that of the model group (hair removal) and the negative group which was treated with PBS. The positive control and SDEVs groups were significantly more effective than the blank and negative control groups. On the 21st day, the hairs in the blank group, positive control group and SDEVs group were almost full-grown, while those in the negative control group were not full-grown. The earlier appearance of gray-to-black skin and visible hair shafts in the SDEV-treated mice suggests that SDEVs may accelerate the transition of hair follicles from telogen to anagen and promote early-stage hair regrowth. In C57BL/6 mice, dorsal skin pigmentation is closely associated with anagen-related melanogenesis and can therefore serve as an indirect indicator of hair-cycle progression. The enhanced hair coverage observed in the SDEVs group on day 14 further supports a potential hair growth-promoting effect. However, because hair coverage in the model and SDEVs groups became comparable by day 21, SDEVs may primarily accelerate the onset and early progression of hair growth rather than increase the final extent of hair coverage. Histological and molecular analyses are required to confirm hair-cycle conversion and clarify the underlying mechanism.

**Fig 7:**
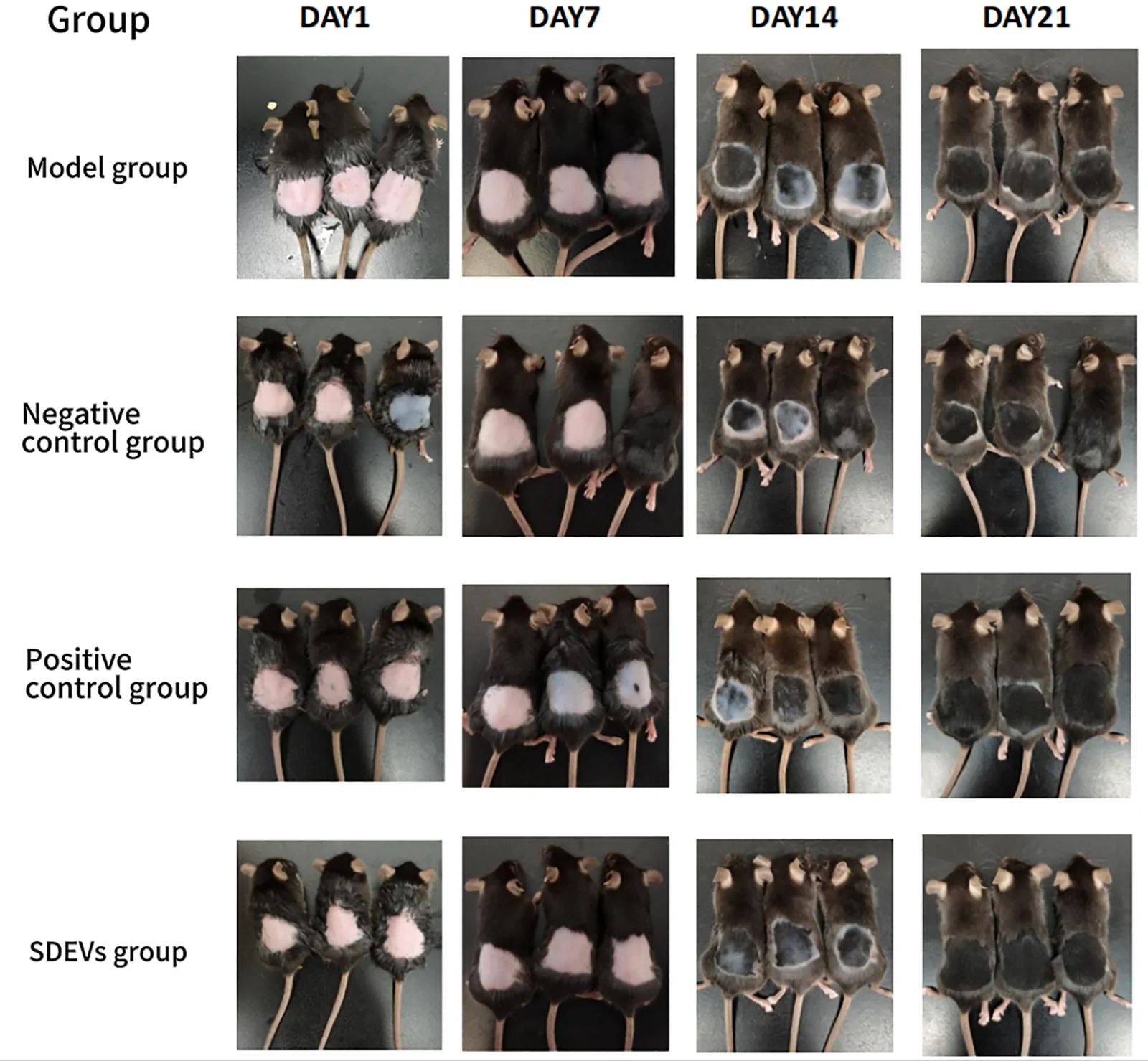
Hair growth of mice in each group after administration.

Skin tissues were collected from the dorsal treatment area and subjected to histological examination using hematoxylin and eosin staining. Compared with the model and negative control groups, the positive control and SDEVs groups exhibited more prominent hair follicles extending into the deep dermal and subcutaneous layers. A greater number of visible hair follicle profiles was also observed in these groups. These morphological characteristics are consistent with more active anagen-stage follicles, suggesting that SDEVs treatment may facilitate the transition of hair follicles from telogen to anagen and promote early hair growth. These histological findings were consistent with the macroscopic hair-growth observations shown in Fig 7 and further support the potential hair growth-promoting effect of SDEVs (Fig 8).

**Fig 8:**
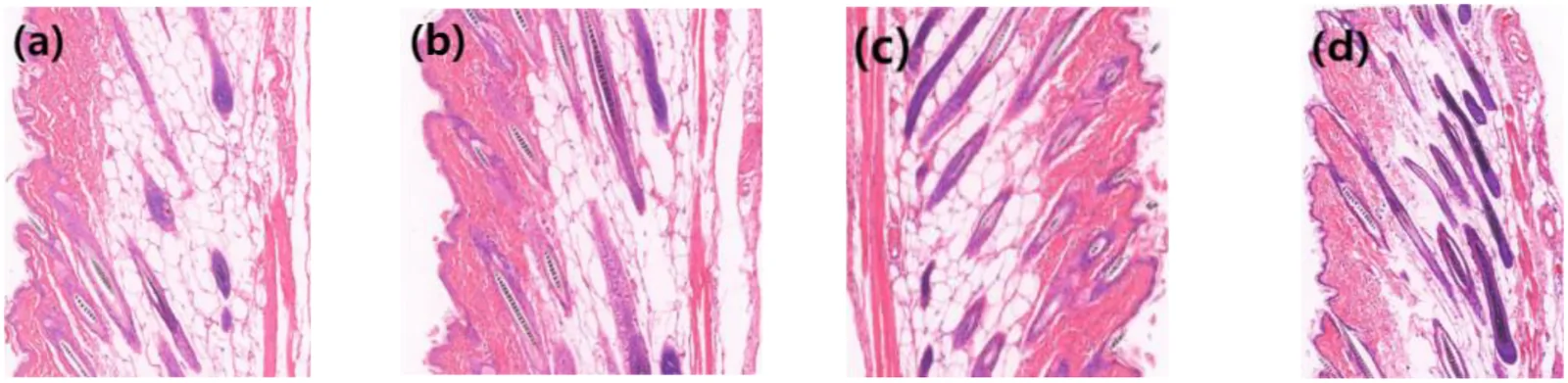
Vertical slices of the skin of the mice in each group (×200) (a) Model group; (b) Negative control group; (c) Positive control group; (d) SDEVs group.

To investigate the potential biological functions of proteins enriched in SDEVs, functional enrichment analyses were performed on the highly abundant proteins. Gene Ontology (GO) analyses were performed on the highly abundant proteins. Gene Ontology (GO) analysis showed that these proteins were significantly enriched in biological processes related to autophagosome assembly, cellular response to hydrogen peroxide, intracellular calcium ion homeostasis, lipid catabolic process, and odontogenesis. Additional enriched terms included antigen processing and presentation, carbon dioxide transport, cardiac myofibril assembly, cellular hyperosmotic response, intracellular water homeostasis, and protein refolding (Fig 9). Collectively, these results indicate that SDEVs proteins are functionally associated with stress adaptation, metabolic regulation, intracellular homeostasis, and membrane-associated biological processes.

**Fig 9:**
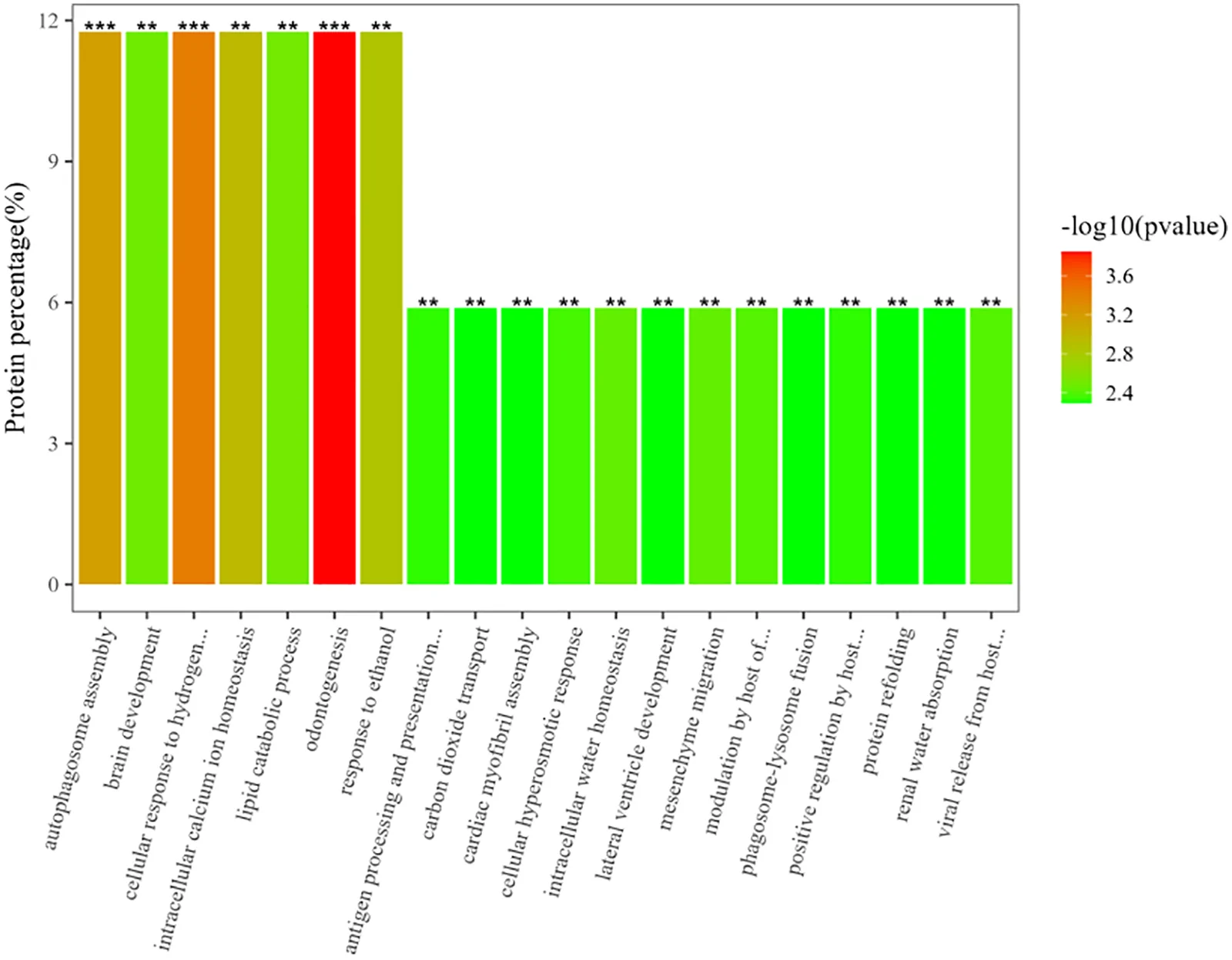
Functional enrichment analysis of highly abundant protein in SDEVs: GO enrichment analysis.

Kyoto Encyclopedia of Genes and Genomes (KEGG) pathway enrichment analysis further revealed that the highly abundant SDEVs proteins were primarily enriched in pathways related to the lysosome and autophagy, with additional enrichment in antigen processing and presentation, endocytosis, estrogen signaling pathway, mitophagy, and phagosome (Fig 10). Among these, lysosome- and autophagy-related pathways showed the strongest enrichment signals, consistent with the GO results and supporting a close association of SDEVs proteins with vesicle trafficking, intracellular degradation, and proteostasis regulation. The enrichment of pathways involved in endocytosis, phagosome formation, and antigen processing further suggests that these vesicles may participate in cargo transport and cellular communication processed after uptake by recipient cells.

**Fig 10:**
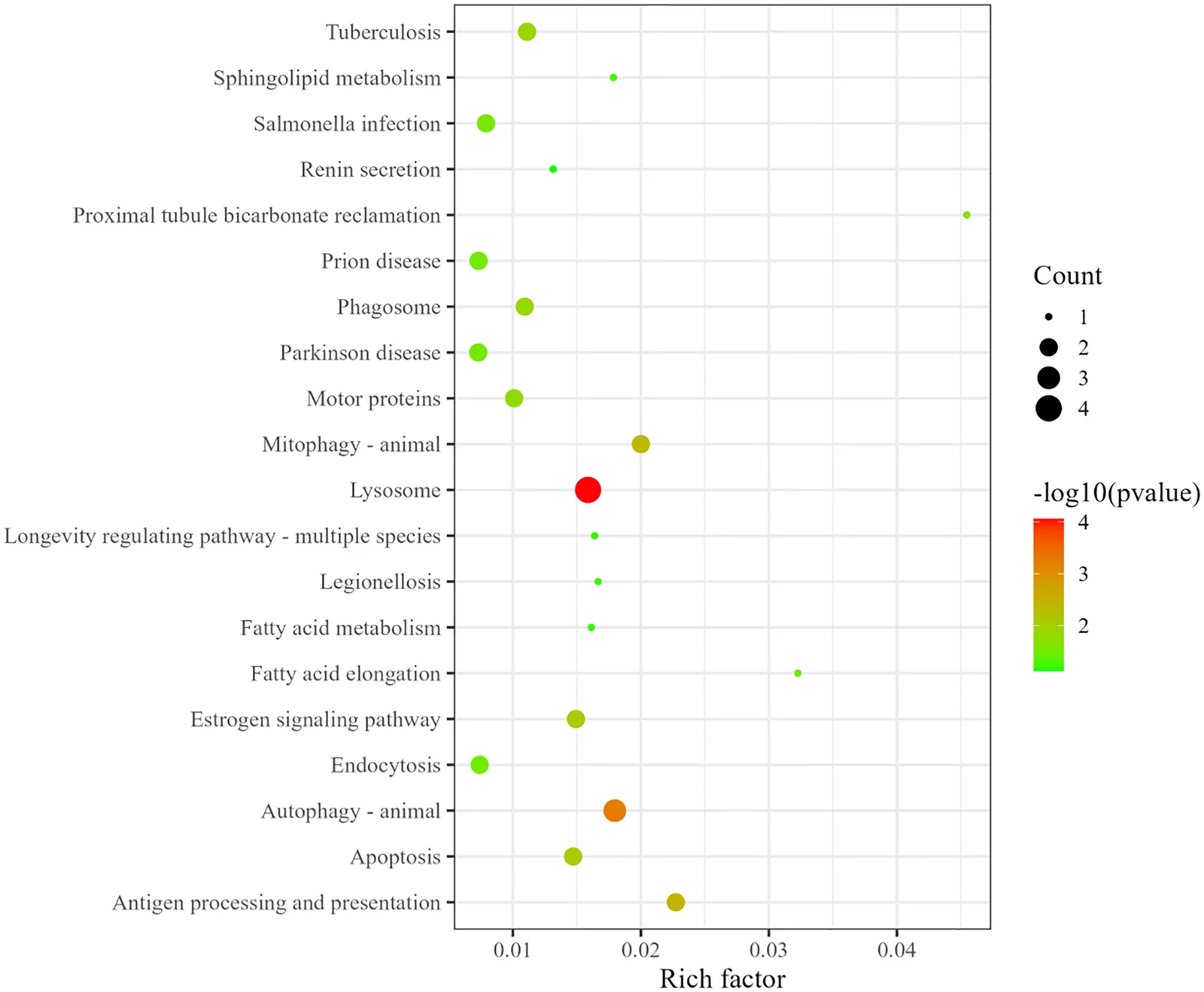
Functional enrichment analysis of highly abundant protein in SDEVs: KEGG enrichment analysis.

## 4. Discussion

The present study suggests that spruce-derived extracellular vesicles (SDEVs) may activate dermal papilla cells (DPCs) and accelerate hair regrowth in depilated C57BL/6 mice. Increased EdU incorporation and Ki-67 positivity provide more direct evidence of DPCs proliferation than the CCK-8 assay. In vivo, earlier dorsal skin darkening and the appearance of visible hair shafts, together with H&E staining showing deeper and more prominent hair follicle structures, suggest that SDEVs may facilitate the transition of hair follicles from telogen to anagen. However, these findings primarily support a potential hair growth-promoting effect of SDEVs and are insufficient to establish a specific therapeutic mechanism.

EVs from different biological sources may act on similar cellular processes, including DPCs activation, cell survival, epithelial-mesenchymal interactions within hair follicles, and anagen initiation. Nevertheless, the bioactive components of SDEVs are unlikely to be identical to those of mammalian EVs. Mammalian EVs can directly carry growth factors, signaling proteins, and regulatory miRNAs involved in hair follicle biology. In contrast, plant-derived EVs may mediate cross-kingdom regulation through plant proteins, lipids, small RNAs, and secondary metabolites. Direct evidence that plant-derived vesicles promote hair growth remains limited. A recent study showed that nanovesicles derived from Polygonum multiflorum roots promoted DPCs growth, increased β-catenin expression and nuclear translocation, and enhanced hair-shaft elongation in ex vivo cultured human hair follicles. These findings are consistent with the DPCs proliferation and hair-regrowth effects observed in the present study. However, that study also did not identify the specific active components responsible for the biological effects of nanovesicles, reflecting a common limitation in research on plant-derived vesicles. The present study extends the potential plant sources of hair growth-promoting vesicles to spruce; however, further experiments are needed to determine whether SDEVs act through mechanisms shared with other plant-derived vesicles.

In the present animal experiment, more pronounced hair regrowth was observed in the SDEVs group on day 14, suggesting that SDEVs may accelerate the initiation and early progression of hair regeneration. In C57BL/6 mice, dorsal skin color changes from pink to gray or black are generally associated with anagen-related melanogenesis. The subsequent appearance of short hair shafts further suggests that the follicles have entered an active growth phase. Therefore, these findings are consistent with earlier anagen entry. However, by day 21, hair regrowth was nearly complete in both the blank control and SDEV groups. The convergence of hair coverage at the endpoint suggests that the major effect of SDEVs may be to shorten the time required for spontaneous hair regrowth after depilation, rather than to increase the final extent of hair coverage. The greater number of hair follicle profiles observed in SDEV-treated skin sections may indicate that more pre-existing follicles entered anagen and became enlarged or extended into deeper subcutaneous tissue.

A limitation of this study is the absence of Western blot validation of SDEVs-associated proteins. Currently, no universally accepted EVs protein markers or validated species-specific antibodies are available for spruce-derived EVs. Therefore, the identity of the isolated vesicles was assessed using complementary morphological and particle-size characterization methods. Further proteomic analysis and the development of antibodies against spruce-specific EV-associated proteins will be required to establish molecular markers for SDEVs. However, this study still needs to be further improved, such as in-depth exploration of the mechanism of the effect on DPCs, and in-depth exploration of the effect of SDEVs on mouse models of drug or androgenetic alopecia.
