# Supplementary material for: Spruce-derived extracellular vesicles (SDEVs) improve hair loss by activating the proliferation of dermal papilla cells
Source: PLoS One. 2026 Nov 6;21(11):e0358041. doi: 10.1371/journal.pone.0358041 (PMC13742931; doi:10.1371/journal.pone.0358041)
Supplement: Supplementary file 1 [file pone.0358041.s002.doc]

**General Response**

We sincerely thank the Editor and Reviewer for their careful evaluation of our manuscript and for providing constructive comments. We have thoroughly examined the manuscript and made comprehensive revisions in response to all the comments. The major revisions include removing the experimental group that was erroneously mentioned in the Results section, rechecking all experimental group assignments throughout the manuscript, supplementing and clarifying the characterization and limitations of SDEVs, adding EdU and Ki-67 proliferation assays, reanalyzing and quantifying the cell proliferation results, substantially rewriting the Discussion section, and systematically improving the language, logic, and formatting of the manuscript. Our detailed responses are provided below.

**Comment 1: The manuscript has several overall deficiencies**

**Reviewer’s comment:**

The manuscript has several deficiencies in its current form. The authors are required to address all the points raised by the reviewer and revise the manuscript accordingly.

**Response:**

We thank the Reviewer for this overall assessment. We acknowledge these concerns and have thoroughly reviewed and revised the manuscript. In addition to addressing each specific issue below, we carefully rechecked the consistency among the experimental groups, Methods, Results, figure legends, and statistical analyses. We also systematically revised the language and structure of the manuscript. All changes have been highlighted in the revised manuscript with tracked changes.

**Comment 2: Erroneous mention of the tea bran alcohol extract group**

**Reviewer’s comment:**

On page 15, the text suddenly introduces a "Tea Bran Alcohol Extract group" ("on the 9th day of administration, the site of the Tea Bran Alcohol Extract group began to turn gray-black"). This group is never mentioned in the Abstract, Introduction, or Methods. This demonstrates a severe lack of quality control without proper scientific integrity checks.

**Response:**

We sincerely apologize for this error. The term“Tea Bran Alcohol Extract group” was an inadvertent wording error left over from the manuscript revision process and does not represent an experimental group in the present study. We have deleted this term and carefully rechecked all experimental group assignments throughout the manuscript against the original experimental records.

We have revised this part of the manuscript as follows:

The skin at the hair removal site of mice (on the first day) appeared light pink, on the 7th day of administration, the site of the positive control group began to turn gray-black. On the 14th day of administration, the back of the mice of each group appeared to have centrally diffused growth of black glossy neonatal hair, and the effect of the positive control group (treaede with minoxidil) and the SDEVs group was significantly higher than that of the model group (hair removal) and the negative control group which was treated with PBS.

We have added additional information to the revised manuscript as follows:

The earlier appearance of gray-to-black skin and visible hair shafts in the SDEV-treated mice suggests that SDEVs may accelerate the transition of hair follicles from telogen to anagen and promote early-stage hair regrowth. In C57BL/6 mice, dorsal skin pigmentation is closely associated with anagen-related melanogenesis and can therefore serve as an indirect indicator of hair-cycle progression. The enhanced hair coverage observed in the SDEVs group on day 14 further supports a potential hair growth-promoting effect. However, because hair coverage in the model and SDEV groups became comparable by day 21, SDEVs may primarily accelerate the onset and early progression of hair growth rather than increase the final extent of hair coverage. Histological and molecular analyses are required to confirm hair-cycle conversion and clarify the underlying mechanism.

**Comment 3: Insufficient molecular characterization of SDEVs**

**Reviewer’s comment:**

The authors claim to have isolated Spruce-Derived Extracellular Vesicles (SDEVs), but the characterization in Section 2.3 is strictly limited to morphology (TEM) and size distribution (NTA). In plant-derived extracellular vesicles (PDEVs), it is an absolute requirement to validate the isolation by assessing specific plant tetraspanin markers, such as TET8 and TET9, via Western Blot or targeted proteomics. Measuring total protein concentration (3.537 mg/mL) is insufficient, as this could reflect co-isolated background proteins or intracellular contaminants from the crude spruce needle juice extraction .

**Response:**

We thank the Reviewer for pointing out the insufficient characterization of SDEVs. We agree that TEM, NTA, and protein quantification alone cannot fully confirm the biogenesis of the isolated particles.

The samples analyzed in this study were spruce-derived extracellular vesicles (SDEVs). At present, sufficiently validated species-specific protein markers and commercially available antibodies for spruce-derived EVs remain limited. TET8 and TET9 have been primarily investigated in model plants such as Arabidopsis thaliana. Their specificity as EV markers in spruce, a gymnosperm species, and the cross-reactivity of currently available antibodies with spruce homologs have not yet been established. Therefore, a negative result obtained using these antibodies could reflect interspecies sequence divergence or insufficient antibody recognition, rather than the absence of SDEVs.

Because specific markers and validated antibodies for SDEVs are currently unavailable, we did not have Western blot results that would be difficult to interpret reliably. We have also explicitly acknowledged in the Discussion that further molecular characterization of SDEVs will require proteomic analysis, identification of candidate spruce EVs-associated proteins, and the development of species-specific antibodies.

**Comment 4: CCK-8 assay cannot directly demonstrate cell proliferation**

**Reviewer’s comment:**

The core claim is that SDEVs "activate the proliferation of Dermal papilla cells". However, the authors relied exclusively on the CCK-8 assay . The CCK-8 assay measures cellular metabolic activity (mitochondrial dehydrogenase activity) rather than direct cell division. Increased absorbance can indicate enhanced cell viability, metabolic upregulation, or cellular hypertrophy, but it does not definitively prove proliferation. To substantiate this claim, direct replication assays (e.g., EdU/BrdU incorporation, Ki-67 staining, or direct cell counting over a time course) are strictly required.

**Response:**

We thank the Reviewer for this important comment. We agree that the CCK-8 assay primarily reflects cellular metabolic activity and the relative number of viable cells, and therefore cannot independently demonstrate cell division.

In accordance with the Reviewer’s suggestion, we performed EdU incorporation and Ki-67 immunofluorescence assays. DPCs were treated with 600 μg/mL SDEVs for 24 or 48 h, after which the proportions of EdU-positive and Ki-67-positive cells were determined. These results provide more direct evidence that SDEVs promote DPC proliferation. The experimental procedures have been added to the Methods section. We have added the corresponding results to the revised manuscript as follows:

EdU incorporation and Ki-67 immunofluorescence assays both showed that SDEVs treatment increased proliferation-related indices in DPCs. In the EdU assay, S-phase cells were labeled with Azide 594. DAPI was used to counterstain cell nuclei and determine total cell number. For Ki-67 immunofluorescence staining, cells were incubated with a rabbit monoclonal anti-Ki-67 antibody followed by an Alexa Fluor® 488-conjugated anti-rabbit IgG agment antibody. Ki-67-positive cells in the G1, S, G2, and M phases are shown in green, whereas quiescent G0-phase cells are Ki-67-negative. The control group received the same DPCs-specific medium without SDEVs. After 24 h of treatment, the proportion of EdU-positive cells increased from 20.0% to 23.5%, while the proportion of Ki-67-positive cells increased from 68.8% to 70.8%. After 48 h of treatment, the proportion of EdU-positive cells increased from 18.9% to 23.5%, and the proportion of Ki-67-positive cells increased from 73.9% to 80.0% (Figure 3). The increase in EdU positivity indicates that a greater proportion of DPCs entered the S phase and underwent DNA synthesis, whereas the increased Ki-67 positivity suggests an increased proportion of cells in an active cell-cycle state. The consistent direction of these two assays indicates that SDEVs may promote cell-cycle progression and proliferative activity in DPCs, with a more pronounced effect observed at 48 h. Compared with the CCK-8 assay, which primarily reflects cellular metabolic activity, the EdU and Ki-67 results provide more direct cellular evidence that SDEVs promote DPCs proliferation.

**Figure 3. Representative confocal images of EdU incorporation and Ki-67 immunofluorescen ce staining in DPCs treated with 600 μg/mL SDEVs for 24 h and 48 h. Azide 594: red, excitation/emission maxima: 590/615 nm, DAPI: blue, excitation/emission maxima: 358/461 nm, Alexa Fluor® 488: excitation/emission maxima: 495/519 nm.**

**Comment 5: The Discussion section is overly superficial**

**Reviewer’s comment:**

The Discussion section is unacceptably brief and superficial. It acts merely as a repetitive summary of the results section rather than a critical scientific evaluation. The authors completely fail to place their findings in the context of existing literature, discuss the molecular mechanisms behind potassium ion channel activation or VEGF upregulation, or address the inherent limitations of their study .

**Response:**

We thank the Reviewer for this comment. We agree that the original Discussion section did not sufficiently explain the significance, possible mechanisms, and limitations of our findings. Therefore, we have comprehensively rewritten the Discussion section.

We have added discussion in the revised manuscript as follows:

The present study suggests that spruce-derived extracellular vesicles (SDEVs) may activate dermal papilla cells (DPCs) and accelerate hair regrowth in depilated C57BL/6 mice. Increased EdU incorporation and Ki-67 positivity provide more direct evidence of DPC proliferation than the CCK-8 assay. In vivo, earlier dorsal skin darkening and the appearance of visible hair shafts, together with H&E staining showing deeper and more prominent hair follicle structures, suggest that SDEVs may facilitate the transition of hair follicles from telogen to anagen. However, these findings primarily support a potential hair growth-promoting effect of SDEVs and are insufficient to establish a specific therapeutic mechanism.

EVs from different biological sources may act on similar cellular processes, including DPC activation, cell survival, epithelial–mesenchymal interactions within hair follicles, and anagen initiation. Nevertheless, the bioactive components of SDEVs are unlikely to be identical to those of mammalian EVs. Mammalian EVs can directly carry growth factors, signaling proteins, and regulatory miRNAs involved in hair follicle biology. In contrast, plant-derived EVs may mediate cross-kingdom regulation through plant proteins, lipids, small RNAs, and secondary metabolites. Direct evidence that plant-derived vesicles promote hair growth remains limited. A recent study showed that nanovesicles derived from Polygonum multiflorum roots promoted DPC growth, increased β-catenin expression and nuclear translocation, and enhanced hair-shaft elongation in ex vivo cultured human hair follicles. These findings are consistent with the DPC proliferation and hair-regrowth effects observed in the present study. However, that study also did not identify the specific active components responsible for the biological effects of nanovesicles, reflecting a common limitation in research on plant-derived vesicles. The present study extends the potential plant sources of hair growth-promoting vesicles to spruce; however, further experiments are needed to determine whether SDEVs act through mechanisms shared with other plant-derived vesicles.

In the present animal experiment, more pronounced hair regrowth was observed in the SDEV group on day 14, suggesting that SDEVs may accelerate the initiation and early progression of hair regeneration. In C57BL/6 mice, dorsal skin color changes from pink to gray or black are generally associated with anagen-related melanogenesis. The subsequent appearance of short hair shafts further suggests that the follicles have entered an active growth phase. Therefore, these findings are consistent with earlier anagen entry. However, by day 21, hair regrowth was nearly complete in both the blank control and SDEV groups. The convergence of hair coverage at the endpoint suggests that the major effect of SDEVs may be to shorten the time required for spontaneous hair regrowth after depilation, rather than to increase the final extent of hair coverage. The greater number of hair follicle profiles observed in SDEV-treated skin sections may indicate that more pre-existing follicles entered anagen and became enlarged or extended into deeper subcutaneous tissue.

A limitation of this study is the absence of Western blot validation of SDEVs-associated proteins. Currently, no universally accepted EVs protein markers or validated species-specific antibodies are available for spruce-derived EVs. Therefore, the identity of the isolated vesicles was assessed using complementary morphological and particle-size characterization methods. Further proteomic analysis and the development of antibodies against spruce-specific EV-associated proteins will be required to establish molecular markers for SDEVs. However, this study still needs to be further improved, such as in-depth exploration of the mechanism of the effect on DPCs, and in-depth exploration of the effect of SDEVs on mouse models of drug or androgenetic alopecia.

**Comment 6: Poor language and writing quality**

**Reviewer’s comment:**

The manuscript is poorly written and organized and requires comprehensive revision.

**Response:**

We acknowledge this concern and have systematically revised the language and structure of the entire manuscript. Specifically, we corrected grammatical and spelling errors, removed repetitive and unclear sentences, standardized the nomenclature for SDEVs, DPCs, and the experimental groups, reorganized the Discussion section to improve its logic and critical analysis, and carefully rechecked the data and group assignments in the figures, figure legends, and main text. We believe that the revised manuscript has been substantially improved in terms of accuracy, readability, and logical coherence.
