# Supplementary material for: Spruce-derived extracellular vesicles (SDEVs) improve hair loss by activating the proliferation of dermal papilla cells
Source: PLoS One. 2026 Nov 6;21(11):e0358041. doi: 10.1371/journal.pone.0358041 (PMC13742931; doi:10.1371/journal.pone.0358041)
Supplement: Peer review history

# Peer review: Decision Letter 0

**DOI:** 10.1371/journal.pone.0358041.r001

**Authors:** Gianpaolo Papaccio

4 Jun 2026

Dear Dr. Zhang,

Thank you for submitting your manuscript to PLOS ONE. After careful consideration, we feel that it has merit but does not fully meet PLOS ONE’s publication criteria as it currently stands. Therefore, we invite you to submit a revised version of the manuscript that addresses the points raised during the review process.

Moreover there is a deficient Vesicle Characterization: The manuscript lacks molecular validation of the isolated vesicles. No expression analysis of plant-specific tetraspanin markers (such as TET8 or TET9) was performed, failing to comply with standard minimal reporting requirements for extracellular vesicle research (MISEV guidelines).

The assertion that SDEVs stimulate DPC proliferation is supported only by CCK-8 metabolic assays Direct proliferation assays (e.g., EdU/BrdU or Ki-67 quantification) are strictly required to substantiate true cell division.

Please submit your revised manuscript by Aug 03 2026 11:59PM. If you will need more time than this to complete your revisions, please reply to this message or contact the journal office at plosone@plos.org. When you're ready to submit your revision, log on to https://www.editorialmanager.com/pone/ and select the 'Submissions Needing Revision' folder to locate your manuscript file.

- A letter that responds to each point raised by the academic editor and reviewer(s). You should upload this letter as a separate file labeled 'Response to Reviewers'.
- A marked-up copy of your manuscript that highlights changes made to the original version. You should upload this as a separate file labeled 'Revised Manuscript with Track Changes'.
- An unmarked version of your revised paper without tracked changes. You should upload this as a separate file labeled 'Manuscript'.

If you would like to make changes to your financial disclosure, please include your updated statement in your cover letter. Guidelines for resubmitting your figure files are available below the reviewer comments at the end of this letter.

If applicable, we recommend that you deposit your laboratory protocols in protocols.io to enhance the reproducibility of your results. Protocols.io assigns your protocol its own identifier (DOI) so that it can be cited independently in the future. For instructions see: https://journals.plos.org/plosone/s/submission-guidelines#loc-laboratory-protocols. Additionally, PLOS ONE offers an option for publishing peer-reviewed Lab Protocol articles, which describe protocols hosted on protocols.io. Read more information on sharing protocols at https://plos.org/protocols?utm_medium=editorial-email&utm_source=authorletters&utm_campaign=protocols.

As the corresponding author, your ORCID iD is verified in the submission system and will appear in the published article. PLOS supports the use of ORCID, and we encourage all coauthors to register for an ORCID iD and use it as well. Please encourage your coauthors to verify their ORCID iD within the submission system before final acceptance, as unverified ORCID iDs will not appear in the published article. *Only*  the individual author can complete the verification step; PLOS staff *cannot*  verify ORCID iDs on behalf of authors.

We look forward to receiving your revised manuscript.

Kind regards,

Gianpaolo Papaccio, M.D., Ph.D.

Academic Editor

PLOS One

Journal Requirements:

When submitting your revision, we need you to address these additional requirements.

1. Please ensure that your manuscript meets PLOS ONE's style requirements, including those for file naming. The PLOS ONE style templates can be found at

https://journals.plos.org/plosone/s/file?id=wjVg/PLOSOne_formatting_sample_main_body.pdf and

https://journals.plos.org/plosone/s/file?id=ba62/PLOSOne_formatting_sample_title_authors_affiliations.pdf

2. We note that the grant information you provided in the ‘Funding Information’ and ‘Financial Disclosure’ sections do not match.

When you resubmit, please ensure that you provide the correct grant numbers for the awards you received for your study in the ‘Funding Information’ section.

3. Thank you for stating the following financial disclosure:

“This study was supported by the National Natural Science Foundation of China (NSFC 81801833) and the Key Program of National Natural Science Foundation of China (Grant No.81830052). “

Please state what role the funders took in the study. If the funders had no role, please state: "The funders had no role in study design, data collection and analysis, decision to publish, or preparation of the manuscript."

If this statement is not correct you must amend it as needed.

Please include this amended Role of Funder statement in your cover letter; we will change the online submission form on your behalf.

4. Thank you for stating the following in the Acknowledgments Section of your manuscript:

“Thanks all the supports from Shanghai Key Laboratory of Molecular Imaging, Shanghai University of Medicine and Health Sciences. This study was supported by the National Natural Science Foundation of China (NSFC 81801833) and the Key Program of National Natural Science Foundation of China (Grant No.81830052).”

We note that you have provided funding information that is not currently declared in your Funding Statement. However, funding information should not appear in the Acknowledgments section or other areas of your manuscript. We will only publish funding information present in the Funding Statement section of the online submission form.

Please remove any funding-related text from the manuscript and let us know how you would like to update your Funding Statement. Currently, your Funding Statement reads as follows:

“The author(s) received no specific funding for this work”

Please include your amended statements within your cover letter; we will change the online submission form on your behalf.

5. When completing the data availability statement of the submission form, you indicated that you will make your data available on acceptance. We strongly recommend all authors decide on a data sharing plan before acceptance, as the process can be lengthy and hold up publication timelines. Please note that, though access restrictions are acceptable now, your entire data will need to be made freely accessible if your manuscript is accepted for publication. This policy applies to all data except where public deposition would breach compliance with the protocol approved by your research ethics board. If you are unable to adhere to our open data policy, please kindly revise your statement to explain your reasoning and we will seek the editor's input on an exemption. Please be assured that, once you have provided your new statement, the assessment of your exemption will not hold up the peer review process.

6. PLOS requires an ORCID iD for the corresponding author in Editorial Manager on papers submitted after December 6th, 2016. Please ensure that you have an ORCID iD and that it is validated in Editorial Manager. To do this, go to ‘Update my Information’ (in the upper left-hand corner of the main menu), and click on the Fetch/Validate link next to the ORCID field. This will take you to the ORCID site and allow you to create a new iD or authenticate a pre-existing iD in Editorial Manager.

7. If the reviewer comments include a recommendation to cite specific previously published works, please review and evaluate these publications to determine whether they are relevant and should be cited. There is no requirement to cite these works unless the editor has indicated otherwise.

[Note: HTML markup is below. Please do not edit.]

Reviewers' comments:

Reviewer's Responses to Questions

**Comments to the Author**

1. Is the manuscript technically sound, and do the data support the conclusions?

Reviewer #1: No

**********

2. Has the statistical analysis been performed appropriately and rigorously?

Reviewer #1: Yes

**********

3. Have the authors made all data underlying the findings in their manuscript fully available?

The PLOS Data policy

Reviewer #1: No

**********

4. Is the manuscript presented in an intelligible fashion and written in standard English?

Reviewer #1: No

**********

Reviewer #1: The manuscript investigates the potential of Spruce-Derived Extracellular Vesicles (SDEVs) to treat hair loss using in vitro dermal papilla cells (DPCs) and in vivo C57BL/6 mouse models. This manuscript is extremely superficial, poorly drafted, and scientifically weak. It suffers from critical text contradictions, severe methodological flaws, non-compliance with international vesicle guidelines, and a very poor discussion section that lacks any real critical analysis. Therefore, the manuscript is completely unsuitable for publication.

On page 15, the text suddenly introduces a "Tea Bran Alcohol Extract group" ("on the 9th day of administration, the site of the Tea Bran Alcohol Extract group began to turn gray-black"). This group is never mentioned in the Abstract, Introduction, or Methods. This demonstrates a severe lack of quality control without proper scientific integrity checks.

The authors claim to have isolated Spruce-Derived Extracellular Vesicles (SDEVs), but the characterization in Section 2.3 is strictly limited to morphology (TEM) and size distribution (NTA). In plant-derived extracellular vesicles (PDEVs), it is an absolute requirement to validate the isolation by assessing specific plant tetraspanin markers, such as TET8 and TET9, via Western Blot or targeted proteomics. Measuring total protein concentration (3.537 mg/mL) is insufficient, as this could reflect co-isolated background proteins or intracellular contaminants from the crude spruce needle juice extraction . The core claim is that SDEVs "activate the proliferation of Dermal papilla cells". However, the authors relied exclusively on the CCK-8 assay . The CCK-8 assay measures cellular metabolic activity (mitochondrial dehydrogenase activity) rather than direct cell division. Increased absorbance can indicate enhanced cell viability, metabolic upregulation, or cellular hypertrophy, but it does not definitively prove proliferation. To substantiate this claim, direct replication assays (e.g., EdU/BrdU incorporation, Ki-67 staining, or direct cell counting over a time course) are strictly required. The Discussion section is unacceptably brief and superficial. It acts merely as a repetitive summary of the results section rather than a critical scientific evaluation. The authors completely fail to place their findings in the context of existing literature, discuss the molecular mechanisms behind potassium ion channel activation or VEGF upregulation, or address the inherent limitations of their study .

The authors state: "All relevant data from this study will be made available upon study completion." (p. 4) This violates PLOS ONE's strict data availability policy, which mandates that all underlying minimal data sets must be fully available at the time of submission.

**********

what does this mean?). If published, this will include your full peer review and any attached files.

If you choose “no”, your identity will remain anonymous but your review may still be made public.

**Do you want your identity to be public for this peer review?**  For information about this choice, including consent withdrawal, please see our Privacy Policy

Reviewer #1: No

**********

[NOTE: If reviewer comments were submitted as an attachment file, they will be attached to this email and accessible via the submission site. Please log into your account, locate the manuscript record, and check for the action link "View Attachments". If this link does not appear, there are no attachment files.]

To ensure your figures meet our technical requirements, please review our figure guidelines: https://journals.plos.org/plosone/s/figures

You may also use PLOS’s free figure tool, NAAS, to help you prepare publication quality figures: https://journals.plos.org/plosone/s/figures#loc-tools-for-figure-preparation.

NAAS will assess whether your figures meet our technical requirements by comparing each figure against our figure specifications.

# Author comment: Author response to Decision Letter 1

**DOI:** 10.1371/journal.pone.0358041.r002

12 Aug 2026

General Response

We sincerely thank the Editor and Reviewer for their careful evaluation of our manuscript and for providing constructive comments. We have thoroughly examined the manuscript and made comprehensive revisions in response to all the comments. The major revisions include removing the experimental group that was erroneously mentioned in the Results section, rechecking all experimental group assignments throughout the manuscript, supplementing and clarifying the characterization and limitations of SDEVs, adding EdU and Ki-67 proliferation assays, reanalyzing and quantifying the cell proliferation results, substantially rewriting the Discussion section, and systematically improving the language, logic, and formatting of the manuscript. Our detailed responses are provided below.

Comment 1: The manuscript has several overall deficiencies

Reviewer’s comment:

The manuscript has several deficiencies in its current form. The authors are required to address all the points raised by the reviewer and revise the manuscript accordingly.

Response:

We thank the Reviewer for this overall assessment. We acknowledge these concerns and have thoroughly reviewed and revised the manuscript. In addition to addressing each specific issue below, we carefully rechecked the consistency among the experimental groups, Methods, Results, figure legends, and statistical analyses. We also systematically revised the language and structure of the manuscript. All changes have been highlighted in the revised manuscript with tracked changes.

Comment 2: Erroneous mention of the tea bran alcohol extract group

Reviewer’s comment:

On page 15, the text suddenly introduces a "Tea Bran Alcohol Extract group" ("on the 9th day of administration, the site of the Tea Bran Alcohol Extract group began to turn gray-black"). This group is never mentioned in the Abstract, Introduction, or Methods. This demonstrates a severe lack of quality control without proper scientific integrity checks.

Response:

We sincerely apologize for this error. The term“Tea Bran Alcohol Extract group” was an inadvertent wording error left over from the manuscript revision process and does not represent an experimental group in the present study. We have deleted this term and carefully rechecked all experimental group assignments throughout the manuscript against the original experimental records.

We have revised this part of the manuscript as follows:

The skin at the hair removal site of mice (on the first day) appeared light pink, on the 7th day of administration, the site of the positive control group began to turn gray-black. On the 14th day of administration, the back of the mice of each group appeared to have centrally diffused growth of black glossy neonatal hair, and the effect of the positive control group (treaede with minoxidil) and the SDEVs group was significantly higher than that of the model group (hair removal) and the negative control group which was treated with PBS.

We have added additional information to the revised manuscript as follows:

The earlier appearance of gray-to-black skin and visible hair shafts in the SDEV-treated mice suggests that SDEVs may accelerate the transition of hair follicles from telogen to anagen and promote early-stage hair regrowth. In C57BL/6 mice, dorsal skin pigmentation is closely associated with anagen-related melanogenesis and can therefore serve as an indirect indicator of hair-cycle progression. The enhanced hair coverage observed in the SDEVs group on day 14 further supports a potential hair growth-promoting effect. However, because hair coverage in the model and SDEV groups became comparable by day 21, SDEVs may primarily accelerate the onset and early progression of hair growth rather than increase the final extent of hair coverage. Histological and molecular analyses are required to confirm hair-cycle conversion and clarify the underlying mechanism.

Comment 3: Insufficient molecular characterization of SDEVs

Reviewer’s comment:

The authors claim to have isolated Spruce-Derived Extracellular Vesicles (SDEVs), but the characterization in Section 2.3 is strictly limited to morphology (TEM) and size distribution (NTA). In plant-derived extracellular vesicles (PDEVs), it is an absolute requirement to validate the isolation by assessing specific plant tetraspanin markers, such as TET8 and TET9, via Western Blot or targeted proteomics. Measuring total protein concentration (3.537 mg/mL) is insufficient, as this could reflect co-isolated background proteins or intracellular contaminants from the crude spruce needle juice extraction .

Response:

We thank the Reviewer for pointing out the insufficient characterization of SDEVs. We agree that TEM, NTA, and protein quantification alone cannot fully confirm the biogenesis of the isolated particles.

The samples analyzed in this study were spruce-derived extracellular vesicles (SDEVs). At present, sufficiently validated species-specific protein markers and commercially available antibodies for spruce-derived EVs remain limited. TET8 and TET9 have been primarily investigated in model plants such as Arabidopsis thaliana. Their specificity as EV markers in spruce, a gymnosperm species, and the cross-reactivity of currently available antibodies with spruce homologs have not yet been established. Therefore, a negative result obtained using these antibodies could reflect interspecies sequence divergence or insufficient antibody recognition, rather than the absence of SDEVs.

Because specific markers and validated antibodies for SDEVs are currently unavailable, we did not have Western blot results that would be difficult to interpret reliably. We have also explicitly acknowledged in the Discussion that further molecular characterization of SDEVs will require proteomic analysis, identification of candidate spruce EVs-associated proteins, and the development of species-specific antibodies.

Comment 4: CCK-8 assay cannot directly demonstrate cell proliferation

Reviewer’s comment:

The core claim is that SDEVs "activate the proliferation of Dermal papilla cells". However, the authors relied exclusively on the CCK-8 assay . The CCK-8 assay measures cellular metabolic activity (mitochondrial dehydrogenase activity) rather than direct cell division. Increased absorbance can indicate enhanced cell viability, metabolic upregulation, or cellular hypertrophy, but it does not definitively prove proliferation. To substantiate this claim, direct replication assays (e.g., EdU/BrdU incorporation, Ki-67 staining, or direct cell counting over a time course) are strictly required.

Response:

We thank the Reviewer for this important comment. We agree that the CCK-8 assay primarily reflects cellular metabolic activity and the relative number of viable cells, and therefore cannot independently demonstrate cell division.

In accordance with the Reviewer’s suggestion, we performed EdU incorporation and Ki-67 immunofluorescence assays. DPCs were treated with 600 μg/mL SDEVs for 24 or 48 h, after which the proportions of EdU-positive and Ki-67-positive cells were determined. These results provide more direct evidence that SDEVs promote DPC proliferation. The experimental procedures have been added to the Methods section. We have added the corresponding results to the revised manuscript as follows:

EdU incorporation and Ki-67 immunofluorescence assays both showed that SDEVs treatment increased proliferation-related indices in DPCs. In the EdU assay, S-phase cells were labeled with Azide 594. DAPI was used to counterstain cell nuclei and determine total cell number. For Ki-67 immunofluorescence staining, cells were incubated with a rabbit monoclonal anti-Ki-67 antibody followed by an Alexa Fluor® 488-conjugated anti-rabbit IgG agment antibody. Ki-67-positive cells in the G1, S, G2, and M phases are shown in green, whereas quiescent G0-phase cells are Ki-67-negative. The control group received the same DPCs-specific medium without SDEVs. After 24 h of treatment, the proportion of EdU-positive cells increased from 20.0% to 23.5%, while the proportion of Ki-67-positive cells increased from 68.8% to 70.8%. After 48 h of treatment, the proportion of EdU-positive cells increased from 18.9% to 23.5%, and the proportion of Ki-67-positive cells increased from 73.9% to 80.0% (Figure 3). The increase in EdU positivity indicates that a greater proportion of DPCs entered the S phase and underwent DNA synthesis, whereas the increased Ki-67 positivity suggests an increased proportion of cells in an active cell-cycle state. The consistent direction of these two assays indicates that SDEVs may promote cell-cycle progression and proliferative activity in DPCs, with a more pronounced effect observed at 48 h. Compared with the CCK-8 assay, which primarily reflects cellular metabolic activity, the EdU and Ki-67 results provide more direct cellular evidence that SDEVs promote DPCs proliferation.

Figure 3. Representative confocal images of EdU incorporation and Ki-67 immunofluorescen ce staining in DPCs treated with 600 μg/mL SDEVs for 24 h and 48 h. Azide 594: red, excitation/emission maxima: 590/615 nm, DAPI: blue, excitation/emission maxima: 358/461 nm, Alexa Fluor® 488: excitation/emission maxima: 495/519 nm.

Comment 5: The Discussion section is overly superficial

Reviewer’s comment:

The Discussion section is unacceptably brief and superficial. It acts merely as a repetitive summary of the results section rather than a critical scientific evaluation. The authors completely fail to place their findings in the context of existing literature, discuss the molecular mechanisms behind potassium ion channel activation or VEGF upregulation, or address the inherent limitations of their study .

Response:

We thank the Reviewer for this comment. We agree that the original Discussion section did not sufficiently explain the significance, possible mechanisms, and limitations of our findings. Therefore, we have comprehensively rewritten the Discussion section.

We have added discussion in the revised manuscript as follows:

The present study suggests that spruce-derived extracellular vesicles (SDEVs) may activate dermal papilla cells (DPCs) and accelerate hair regrowth in depilated C57BL/6 mice. Increased EdU incorporation and Ki-67 positivity provide more direct evidence of DPC proliferation than the CCK-8 assay. In vivo, earlier dorsal skin darkening and the appearance of visible hair shafts, together with H&E staining showing deeper and more prominent hair follicle structures, suggest that SDEVs may facilitate the transition of hair follicles from telogen to anagen. However, these findings primarily support a potential hair growth-promoting effect of SDEVs and are insufficient to establish a specific therapeutic mechanism.

EVs from different biological sources may act on similar cellular processes, including DPC activation, cell survival, epithelial–mesenchymal interactions within hair follicles, and anagen initiation. Nevertheless, the bioactive components of SDEVs are unlikely to be identical to those of mammalian EVs. Mammalian EVs can directly carry growth factors, signaling proteins, and regulatory miRNAs involved in hair follicle biology. In contrast, plant-derived EVs may mediate cross-kingdom regulation through plant proteins, lipids, small RNAs, and secondary metabolites. Direct evidence that plant-derived vesicles promote hair growth remains limited. A recent study showed that nanovesicles derived from Polygonum multiflorum roots promoted DPC growth, increased β-catenin expression and nuclear translocation, and enhanced hair-shaft elongation in ex vivo cultured human hair follicles. These findings are consistent with the DPC proliferation and hair-regrowth effects observed in the present study. However, that study also did not identify the specific active components responsible for the biological effects of nanovesicles, reflecting a common limitation in research on plant-derived vesicles. The present study extends the potential plant sources of hair growth-promoting vesicles to spruce; however, further experiments are needed to determine whether SDEVs act through mechanisms shared with other plant-derived vesicles.

In the present animal experiment, more pronounced hair regrowth was observed in the SDEV group on day 14, suggesting that SDEVs may accelerate the initiation and early progression of hair regeneration. In C57BL/6 mice, dorsal skin color changes from pink to gray or black are generally associated with anagen-related melanogenesis. The subsequent appearance of short hair shafts further suggests that the follicles have entered an active growth phase. Therefore, these findings are consistent with earlier anagen entry. However, by day 21, hair regrowth was nearly complete in both the blank control and SDEV groups. The convergence of hair coverage at the endpoint suggests that the major effect of SDEVs may be to shorten the time required for spontaneous hair regrowth after depilation, rather than to increase the final extent of hair coverage. The greater number of hair follicle profiles observed in SDEV-treated skin sections may indicate that more pre-existing follicles entered anagen and became enlarged or extended into deeper subcutaneous tissue.

A limitation of this study is the absence of Western blot validation of SDEVs-associated proteins. Currently, no universally accepted EVs protein markers or validated species-specific antibodies are available for spruce-derived EVs. Therefore, the identity of the isolated vesicles was assessed using complementary morphological and particle-size characterization methods. Further proteomic analysis and the development of antibodies against spruce-specific EV-associated proteins will be required to establish molecular markers for SDEVs. However, this study still needs to be further improved, such as in-depth exploration of the mechanism of the effect on DPCs, and in-depth exploration of the effect of SDEVs on mouse models of drug or androgenetic alopecia.

Comment 6: Poor language and writing quality

Reviewer’s comment:

The manuscript is poorly written and organized and requires comprehensive revision.

Response:

We acknowledge this concern and have systematically revised the language and structure of the entire manuscript. Specifically, we corrected grammatical and spelling errors, removed repetitive and unclear sentences, standardized the nomenclature for SDEVs, DPCs, and the experimental groups, reorganized the Discussion section to improve its logic and critical analysis, and carefully rechecked the data and group assignments in the figures, figure legends, and main text. We believe that the revised manuscript has been substantially improved in terms of accuracy, readability, and logical coherence.

Attachment Submitted filename: Response to Reviewers.doc

# Peer review: Decision Letter 1

**DOI:** 10.1371/journal.pone.0358041.r003

**Authors:** Wenguo Cui

26 Aug 2026

Spruce-Derived Extracellular Vesicles (SDEVs) improve hair loss by activating the proliferation of dermal papilla cells

PONE-D-26-22571R1

Dear Dr. Zhang,

We’re pleased to inform you that your manuscript has been judged scientifically suitable for publication and will be formally accepted for publication once it meets all outstanding technical requirements.

Within one week, you’ll receive an e-mail detailing the required amendments. When these have been addressed, you’ll receive a formal acceptance letter and your manuscript will be scheduled for publication.

An invoice will be generated when your article is formally accepted. Please note, if your institution has a publishing partnership with PLOS and your article meets the relevant criteria, all or part of your publication costs will be covered. Please make sure your user information is up-to-date by logging into Editorial Manager at Editorial Manager® and clicking the ‘Update My Information' link at the top of the page. For questions related to billing, please contact billing support.

If your institution or institutions have a press office, please notify them about your upcoming paper to help maximize its impact. If they’ll be preparing press materials, please inform our press team as soon as possible -- no later than 48 hours after receiving the formal acceptance. Your manuscript will remain under strict press embargo until 2 pm Eastern Time on the date of publication. For more information, please contact onepress@plos.org.

Kind regards,

Wenguo Cui, Ph.D

Academic Editor

PLOS One

Additional Editor Comments (optional):

Reviewers' comments:

Reviewer's Responses to Questions

**Comments to the Author**

Reviewer #1: All comments have been addressed

**********

2. Is the manuscript technically sound, and do the data support the conclusions?

Reviewer #1: Yes

**********

3. Has the statistical analysis been performed appropriately and rigorously?

Reviewer #1: Yes

**********

4. Have the authors made all data underlying the findings in their manuscript fully available?

The PLOS Data policy

Reviewer #1: Yes

**********

5. Is the manuscript presented in an intelligible fashion and written in standard English?

Reviewer #1: Yes

**********

Reviewer #1: The revisions introduced in this version have vastly improved the manuscript's quality and scientific rigor. By including the EdU and Ki-67 assays, the authors successfully provided the direct evidence needed to support their proliferation claims. Additionally, removing the experimental group inconsistencies, restructuring the Discussion, and upgrading the language have significantly enhanced the clarity and cohesion of the study.

**********

what does this mean?). If published, this will include your full peer review and any attached files.

If you choose “no”, your identity will remain anonymous but your review may still be made public.

**Do you want your identity to be public for this peer review?**  For information about this choice, including consent withdrawal, please see our Privacy Policy

Reviewer #1: No

**********

# Editor report: Acceptance letter

**DOI:** 10.1371/journal.pone.0358041.r004

**Authors:** Wenguo Cui

PONE-D-26-22571R1

PLOS One

Dear Dr. Zhang,

I'm pleased to inform you that your manuscript has been deemed suitable for publication in PLOS One. Congratulations! Your manuscript is now being handed over to our production team.

At this stage, our production department will prepare your paper for publication. This includes ensuring the following:

* All references, tables, and figures are properly cited

* All relevant supporting information is included in the manuscript submission,

* There are no issues that prevent the paper from being properly typeset

You will receive further instructions from the production team, including instructions on how to review your proof when it is ready. Please keep in mind that we are working through a large volume of accepted articles, so please give us a few days to review your paper and let you know the next and final steps.

Lastly, if your institution or institutions have a press office, please let them know about your upcoming paper now to help maximize its impact. If they'll be preparing press materials, please inform our press team within the next 48 hours. Your manuscript will remain under strict press embargo until 2 pm Eastern Time on the date of publication. For more information, please contact onepress@plos.org.

You will receive an invoice from PLOS for your publication fee after your manuscript has reached the completed accept phase. If you receive an email requesting payment before acceptance or for any other service, this may be a phishing scheme. Learn how to identify phishing emails and protect your accounts at https://explore.plos.org/phishing.

If we can help with anything else, please email us at customercare@plos.org.

Thank you for submitting your work to PLOS One and supporting open access.

Kind regards,

PLOS One Editorial Office Staff

on behalf of

Professor Wenguo Cui

Academic Editor

PLOS One
